# Heterogeneity in patterns of helminth infections across populations of mountain gorillas (*Gorilla beringei beringei*)

**DOI:** 10.1038/s41598-021-89283-4

**Published:** 2021-05-25

**Authors:** Klara J. Petrželková, Carine Uwamahoro, Barbora Pafčo, Barbora Červená, Peter Samaš, Antoine Mudakikwa, Richard Muvunyi, Prosper Uwingeli, Kirsten Gilardi, Julius Nziza, Jean Bosco Noheri, Winnie Eckardt, Felix Ndagijimana, Benard Ssebide, Ricky Okwirokello, Fred Nizeyimana, Eddy Kambale Syaluha, Gaspard Nzayisenga, Luis Flores Girón, Méthode Bahizi, Adrien Emile Ntwari, Jean-Paul Lukusa, Jean Claude Tumushime, Damien Mangura, Jeff Mapilanga, Arthur Kalonji, Robert Aruho, Anna Stryková, Zuzana Tehlarová, Rita Cameira, Linda Lowenstine, Jan Šlapeta, Dušan Romportl, Nicola Ferrari, Michael Cranfield, David Modrý

**Affiliations:** 1 Institute of Vertebrate Biology, The Czech Academy of Sciences, Brno, Czech Republic; 2 Institute of Parasitology, Biology Centre, The Czech Academy of Sciences, Ceske Budejovice, Czech Republic; 3 Liberec Zoo, Liberec, Czech Republic; 4 Dian Fossey Gorilla Fund, Musanze, Rwanda; 5 Department of Pathology and Parasitology, Faculty of Veterinary Medicine, University of Veterinary Sciences Brno, Brno, Czech Republic; 6 Rwanda Development Board, Kigali, Rwanda; 7 Gorilla Doctors (MGVP, Inc.), Davis, CA USA; 8 Centre de Recherche en Sciences Naturelles de Lwiro, Lwiro, Democratic Republic of Congo; 9 Lwiro Primate Rehabilitation Center, Democratic Republic of Congo and Ivan Carter Wildlife Conservation Alliance, Orlando, FL USA; 10 Institut Congolais pour la Conservation de la Nature, Kinshasa, Democratic Republic of Congo; 11 Institut Congolais pour la Conservation de la Nature, Parc National de Kahuzi Biega, Bukavu, Democratic Republic of Congo; 12 Uganda Wildlife Authority, Kampala, Uganda; 13 School of Veterinary Medicine, University of California Davis, Davis, CA USA; 14 Sydney School of Veterinary Science, Faculty of Science, University of Sydney, Sydney, Australia; 15 Department of Physical Geography and Geoecology, Faculty of Science, Charles University, Prague, Czech Republic; 16 Department of Veterinary Medicine, Università degli Studi di Milano, Milan, Italy; 17 Research Center Epidemiology and Molecular Surveillance of Infections ‘‘EpiSoMI’’, Università degli Studi di Milano, Milan, Italy; 18 Department of Botany and Zoology, Faculty of Science, Masaryk University, Brno, Czech Republic

**Keywords:** Ecology, Conservation biology, Parasitology

## Abstract

Conservation efforts have led to the recovery of the endangered mountain gorilla populations. Due to their limited potential for spatial expansion, population densities increased, which may alter the epidemiology of infectious diseases. Recently, clinical gastrointestinal illnesses linked to helminth infections have been recorded in both gorilla populations. To understand drivers and patterns of helminth infections we quantified strongylid and tapeworm infections across both Virunga Massif and Bwindi populations using fecal egg counts. We assessed the impact of age, sex, group size, season and spatial differences used as a proxy, which reflects observed variation in the occurrence of gastrointestinal problems, vegetation types, gorilla subpopulation growth and associated social structure on helminth infections. We revealed striking geographic differences in strongylid infections with higher egg counts mostly in areas with high occurrences of gastrointestinal disease. Increased helminth egg counts were also associated with decreasing group size in some areas. Observed spatial differences may reflect mutual effects of variations in subpopulation growth rates, gorilla social structure, and vegetation associated with altitude across mountain gorilla habitat. Helminth infection intensities in Virunga gorillas were lowest in the youngest and the oldest animals. Elucidating parasite infection patterns of endangered species with low genetic diversity is crucial for their conservation management.

## Introduction

Endangered mountain gorillas (*Gorilla beringei beringei*) live in two populations and entirely within protected areas: one ranging within the Virunga Massif (VM) spanning the borders of Rwanda, Uganda and the Democratic Republic of the Congo (DRC), and the second ranging in the Bwindi-Sarambwe ecosystem in Uganda and DRC. The VM population declined dramatically in 1970s^1^, but the numbers stabilized in the 1980s, and since then the population has been steadily increasing with annual growth rates around 3–4% between 2003 and 2016^2,3^. However, this increase has been almost entirely attributed to the growth rate in habituated groups^2–4^ due to the “extreme conservation” measures these groups benefit from, such as daily group monitoring and protection, veterinary interventions and anti-poaching patrols^5^. Moreover, the population increase was not observed uniformly across VM, which may be due to varying ecological conditions that are linked to different habitat types^3,4,6,7^. We have less information about the history of the Bwindi-Sarambwe population dynamics. Robbins et al.^8^ previously indicated that this second mountain gorilla population showed little or no growth before 2009 in comparison to VM population, but subsequent censuses in 2011 and 2018 detected growth of the population^9,10^.

Although both mountain gorilla populations are growing, their potential spatial expansion is extremely limited due to high human densities adjacent to protected areas inhabited by mountain gorillas. Consequently, increasing numbers of gorillas in both populations are likely to result in rising individual and group densities at least in some areas. The VM population has already exceeded the previously predicted carrying capacity of the environment^11^, as defined by the maximum number of animals an area will support based on resources^9^. In addition, between Mt. Karisimbi and Mt. Visoke (aka Bisoke) in the VM, where some of the highest growth rates of gorilla numbers occurred^3,4^, the mountain gorillas experienced major changes in the social structure leading locally to a threefold increase in group densities. Namely, three stable multi‐male groups, which have been steadily growing from the mid‐1990s until 2006, underwent a series of group fissions and formation of smaller groups including multiple one-male groups^12,13^. As a result, there has been an increase in home range overlap, a reduction in home range areas used exclusively by a single group and annual intergroup encounter rates tripled in this area^13^. In addition, small groups, which are often one-male groups, may experience higher stress levels than large groups, which are often multi-male groups, because they have higher home range overlaps with neighboring groups, inter-group encounter rates, and are more vulnerable to infanticide by external males^14–17^. All those observed changes in population dynamics, social structure, and habitat use may be altering stress levels and cause changes in pathogen epidemiology with subsequent health problems^12,18,19^.

Parasites influence the dynamics of free-living animal populations^20,21^ through their impact on host survival^22^ or reproduction^4^. The severity of parasite-induced diseases often depends on the intensity of infection (number of parasite individuals in the host animal)^23^. Identification of factors affecting parasite infections and their intensities as well as resulting patterns of parasite infections across host populations is essential for optimizing effectiveness of parasite control strategies and management of wildlife, particularly for species of conservation concerns^24^. For soil-transmitted parasites, host population density and habitat use play a central role in the host-parasite interaction by increasing the probability that parasite infectious stages will be ingested by a host^25^. Data from 6670 individual hosts representing 19 mammalian species showed a strong positive correlation between the host’s population density and average gastrointestinal (GIT) strongylid nematode infection intensity^26^. In non-human primates this phenomenon has been demonstrated in fragmented populations, where host densities may increase due to restricted available habitat (e.g.^27,28^). In addition, host habitat characteristics can alter the risk of helminth infections^29^. For example, plant species composition, the leaf form of plants^30^, temperature, and humidity^31,32^ are known to influence helminth larval abundance, development and their migratory behavior^29^. Moreover, it is generally accepted that parasite abundance decreases with elevation^33^, but it is not always true across all parasite taxa. For example, the survival of *Cooperia* and *Ostertagia* strongylids in livestock is optimized for cooler weather due to larval inhibition and thus the infection risk increases at higher elevations^34^. Seasonal differences in helminths (especially strongylid nematodes) are commonly observed in non-human primates (e.g.^35–38^). However, some studies reported highest infection intensities in the wet, while others in the dry season. The impact of host factors, such as sex and age, on parasite infections has also been well documented in non-human primates (e.g.^38,39^). It is widely accepted that males of vertebrate hosts tend to exhibit higher rates of parasitism and disease than females^40–42^, with several main factors claimed to cause sex differences in parasitism: body size dimorphism with possibly larger nutritional requirements in the larger sex, social relationship, diet, habitat and hormones^43^. Three theoretical models are generally used to describe the host age–infection relationship in parasites; Type I (linear) and Type II (asymptotic) relationships are thought to occur in the absence of an effective host immune response and instead depend on immigration-death processes linked to parasite transmission and longevity^44^. Type III (convex) associations have been suggested to indicate adaptive immunity to parasites^45^ and have been reported in non-human primates^36^.

Gastrointestinal helminthiases are typically asymptomatic in wild non-human primates, but host factors like immune status, or extrinsic factors, such as habitat loss, fragmentation and other anthropogenic pressures, can alter the transmission dynamics of helminths and increase host susceptibility, which may exacerbate negative effects of infections on health^46,47^. However, clinical diseases caused by parasites have been observed in mountain gorillas; post-mortem examinations of 60 gorillas from 1985 to 2007 (Mountain Gorilla Veterinary Project [MGVP], publication in review) revealed histopathologic evidence for enteritis in more than 50% of cases, gastritis in more than 35% of cases, and colitis in more than 25% of cases. More recently (2013–2017), several cases of severe gastritis have been diagnosed in VM in Rwanda, particularly in adult gorillas ranging between Mt. Karisimbi and Mt. Visoke, with most cases involving young silverbacks (MGVP, data on file). Trichostrongylid nematodes have been observed in the stomach and small intestines in these cases and the histopathology of observed stomach lesions are typical of those caused by nematodes in a range of domestic and wild mammals, including non-human primates^48–50^. In Bwindi Impenetrable National Park (BINP), the Ugandan part of the Bwindi-Sarambwe ecosystem, similar alarming observations indicating change in parasitic infections have been recorded (MGVP, data on file). Many gorillas have exhibited weight loss, declining body condition and poor haircoats in recent years, and in severe cases (n > 10) anthelmintic treatment were administered to sick gorillas in BINP resulting in marked clinical improvement. Anoplocephalid tapeworms have also been commonly observed during necropsies in gorillas from both VM and BINP, and intensities of infection have been high in some animals (e.g., > 100 tapeworms in one animal) (MGVP, data on file).

**Figure 1:**
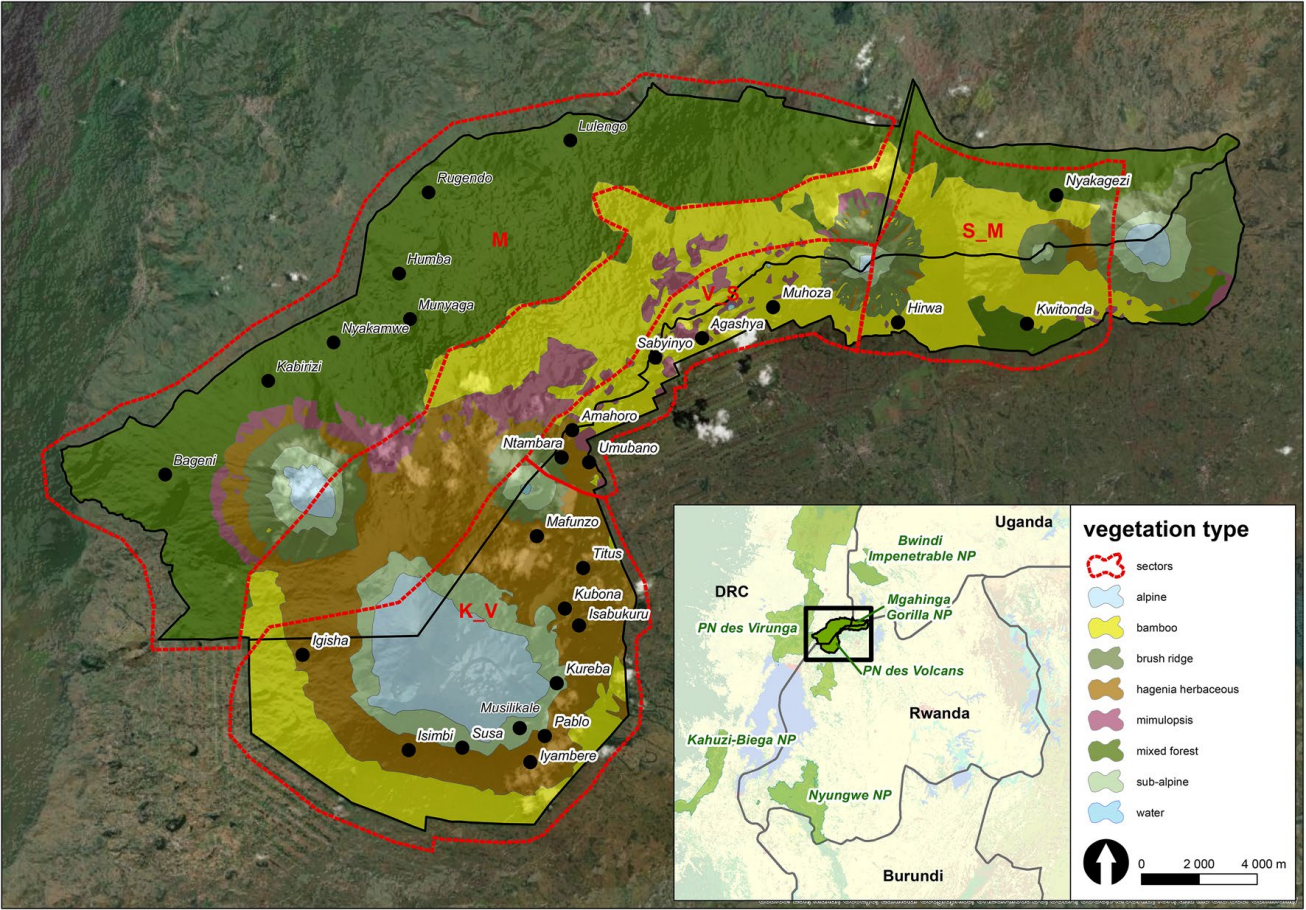
Division of the Virunga Massif into four areas according to volcano positions (M: Mikeno, Virunga National Park; K_V: Karisimbi–Visoke, Volcanoes National Park; V_S: Visoke–Sabyinyo, Volcanoes National Park; S_M: Sabyinyo–Muhavura, Mgahinga Gorilla National Park, Volcanoes National Park) with vegetation zones (based on Robbins et al.^5^) and distribution of the studied groups. Map was created using ArcGIS Desktop 10.6 (ESRI 2019. ArcGIS Desktop: Release 10.6. Redlands, CA: Environmental Systems Research Institute; esri.com). Vegetation data were adopted according to Robbins et al.^5^; boundaries of protected areas were derived from ProtectedPlanet.net database.

Although parasites of mountain gorillas have been studied for almost 100 years^51^, surveys of the spatio-temporal pattern of parasite infections over the entire geographic distribution of mountain gorillas are lacking (for basic studies covering gorilla subpopulations see e.g. ^38,52–54^). We conducted an extensive species-wide survey of helminth infections across the VM and BINP populations and across different seasons to uncover the drivers and patterns of helminth infections and provide a comprehensive foundation for future assessments of the impact of these parasites on gorilla population dynamics. We utilized samples collected from night nests across both populations and samples from individually-identified gorillas from five social groups in VM in Rwanda that experienced differing levels of strongylid infections based on a small-scale pilot study using the same methods applied in this larger-scale study. We divided the VM into four areas according to volcano positions (modification of Weber and Vedder^1^, Fig. 1). This geographic division allowed for a comparison of areas with differences in the occurrence of parasite-related GIT disease, as well as differences in vegetation types, and in historical subpopulation growth and associated current social structure across mountain gorilla distribution range^55,56^. The areas were namely: Karisimbi–Visoke (K_V), Visoke–Sabyinyo (V_S), Sabyinyo–Muhavura (aka Muhabura) (S_M), Mikeno (M). The entirety of BINP was considered as one area. We then investigated the impact of area, sampling period (season), group size and host factors (sex, age) on helminth infections.

In the samples collected from the night nests, we predict that the helminth infections (quantified as eggs per gram in fecal samples; hereafter EPG or egg counts) are higher in the areas with a high occurrence of GIT problems (K_V) than in areas where less or no GIT problems were reported (M, S_M). We predicted that the helminth infections are higher in silverback gorillas in comparison to other age/sex classes and higher in smaller gorilla groups than in larger groups as smaller groups may experience more stress. We further predicted that these effects of age/sex class and group size on the helminth infections are stronger in the areas with higher occurrence of GIT problems and historically higher subpopulation growth. In individually sampled gorillas we predicted that the helminth infections are higher in males than in females, change with age following the convex pattern, and differ between groups as our small-scale pilot project involving five groups suggested. We predicted that age and sex effects are more pronounced in groups with higher helminth egg counts and that convex pattern of egg counts is more pronounced in males than females. In both night nest and individual samples we expect differences in helminth infections between sampling periods, which correspond with different seasons and predicted higher egg counts in the wet season^38^.

## Results

We used three different datasets to test our hypotheses aiming to explain drivers and patterns of helminth infections across five subpopulations (K_V, V_S, S_M, M, BINP) of the mountain gorillas (Fig. 1, Table 1 and Table S1, for details see “Material and methods”). Dataset 1 included nest samples collected across the whole VM and BINP during a single period (n = 392) and was used to investigate the effects of area, age/sex class and group size. Dataset 2 was composed of nest samples but restricted to VM covering two periods (January/February and September/October 2018) (n = 491) and used to examine the effect of area, age/sex class, group size and sampling period (season). Dataset 3 with samples from individually identified gorillas from selected groups in VM collected across two periods (May/June 2018 and September/October 2018) (n = 450) was used to investigate the effects of group identity, sex, exact age^2^ and sampling period (season).

**Table 1:** Sampling design by area (with a national park and country), sampling period and its corresponding season and sample type (for more detailed information about sampled groups, see the Supplementary Table S1).

Park	Area (Weber & Vedder, 1983)^1^	Sampling period (2018)	Season	Sample type
VoNP, Rwanda	Karisimbi–Visoke (K_V) Visoke–Sabyinyo (V_S) Sabyinyo–Muhavura (S_M)	January/February May/June September/October	Dry Boundary wet/dry Wet	Nest Individual Nest + individual
ViNP, DRC	Mikeno (M)	January/February September/October	Dry Wet	Nest Nest
MGNP, Uganda	Sabyinyo–Muhavura (S_M)	September/October	Wet	Nest
BINP, Uganda	BINP	September/October	Wet	Nest

*VoNP* Volcanoes National Park in Rwanda, *ViNP* Virunga National Park in Democratic Republic of the Congo, *MGNP* Mgahinga Gorilla National Park in Uganda, *BINP* Bwindi Impenetrable National Park in Uganda.

**Figure 2:**
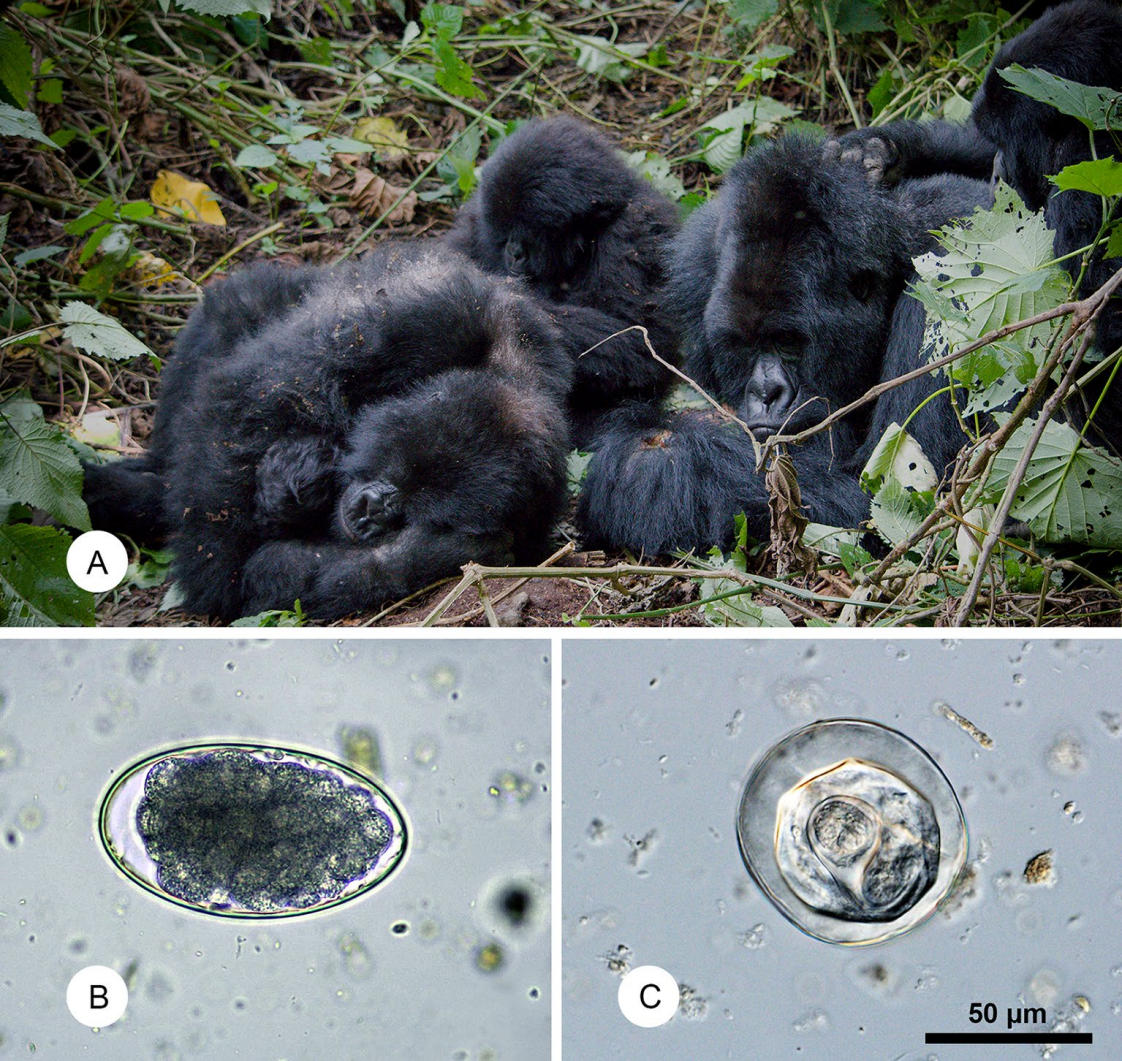
Examined host and their parasites quantified in the study: (**A**) resting group of mountain gorillas in Virunga Massif (Virunga National Park, Democratic Republic of the Congo); (**B**) egg of strongylid nematode; (**C**) egg of anoplocephalid tapeworm; both microphotographs in the same scale.

We observed helminth eggs in the majority of examined gorilla fecal samples (Fig. 2): in dataset 1, the prevalence of the strongylids was 97% (median EPG = 480; range 0–10,050), while the prevalence of the tapeworms was 88% (median EPG = 188; range 0–5775). For the information on strongylid and tapeworm infections in datasets 2 and 3 see Table S1.

### Strongylid infections

**Figure 3:**
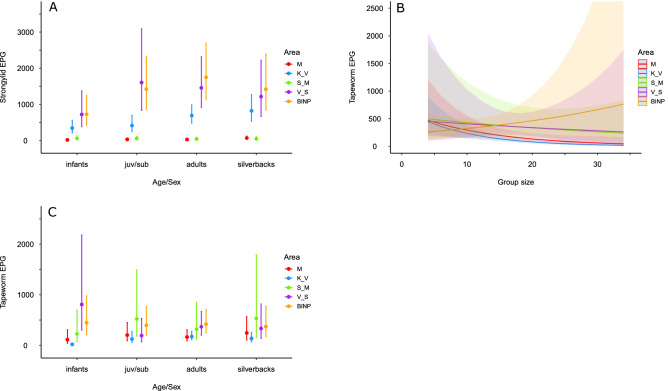
Models’ predicted mean values with 95% confidence intervals for the factors affecting helminth infections in mountain gorillas in Virunga Massif and Bwindi Impenetrable National Park (dataset 1). (**A**) strongylid eggs per gram (EPG)—sampling area and age/sex class effects, (**B**) tapeworm EPG—sampling area and group size effects, (**C**) tapeworm EPG—sampling area and age/sex class effects. juv/sub: juvenile/subadult class; M: Mikeno (Virunga National Park); K_V: Karisimbi–Visoke (Volcanoes National Park), V_S: Visoke–Sabyinyo (Volcanoes National Park), S_M: Sabyinyo–Muhavura (Mgahinga Gorilla National Park, Volcanoes National Park).

In dataset 1, we detected an interactive effect of area and age/sex class (Wald’s Chi-square = 26.7, df = 12, p = 0.008; Table 2) with no clear pattern in M and S_M areas, while in other areas the infants had lower EPG values than non-infant classes (Fig. 3A). M and S_M areas had similar EPG values (Wald’s Chi-square = 1.7, df = 1, p = 0.19) and both had lower EPG values than the other areas (Wald’s Chi-square test, all p < 0.001; Fig. 3A, Table S1). Area K_V had lower EPG values than V_S and BINP areas (Wald’s Chi-square test, both p < 0.01), while the V_S and BINP had similar infection rates (Wald’s Chi-square = 0.1, df = 1, p = 0.92). Infants had lower EPG values than any other age/sex classes (Wald’s Chi-square test, all p < 0.01), while no differences were observed among other sex/age classes (Fig. 3A, Table S1).

**Table 2:** Factors influencing helminth infections in Virunga Massif and Bwindi Impenetrable National Park (dataset 1: nest samples from all national parks).

Effect	*d.f.*	Strongylids	*P value*	Tapeworms	*P value*
		*Chi_square*		*Chi_square*	
Area	4	264.6	< 0.001	19.4	< 0.001
Age/sex	3	33.9	< 0.001	8.4	0.04
Group size	1	0.01	0.91	14.4	< 0.001
Area: age/sex	12	26.7	0.008	32.1	0.001
Area: group size	4	2.7	0.61	10.8	0.03

**Figure 4:**
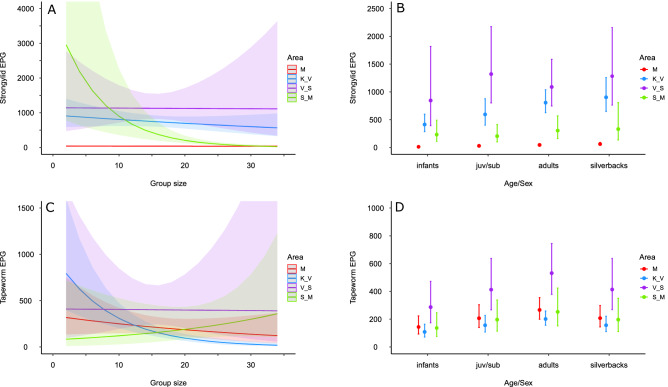
Models’ predicted mean values with 95% confidence intervals for the factors affecting helminth infections in mountain gorillas in Virunga Massif in two periods (dataset 2). (**A**) strongylid egg per gram (EPG)—sampling area and group size effects, (**B**) strongylid EPG—sampling area and age/sex class effects, (**C**) tapeworm EPG—sampling area and group size effects, (**D**) tapeworm EPG—sampling area and age/sex class effects. juv/sub: juvenile/subadult class; M: Mikeno (Virunga National Park); K_V: Karisimbi–Visoke (Volcanoes National Park), V_S: Visoke–Sabyiniyo (Volcanoes National Park), S_M: Sabyinyo–Muhavura (Volcanoes National Park).

In dataset 2, we found an interactive effect of area and group size (Wald’s Chi-square = 20.0, df = 3, p < 0.001; Table 3). The EPG values decreased with increasing group size in the S_M area (estimate =  − 0.14 ± 0.03; z =  − 5.0, p < 0.001) but there was no relationship in other areas (Fig. 4A). All the areas differed from each other (Wald’s Chi-square test, all p < 0.04; Fig. 4A,B, Table 3). EPG values were lower in infants compared to other age/sex classes (Wald’s Chi-square test, all p < 0.002), while no differences were observed among other age/sex classes (Fig. 4B, Table S1).

**Table 3:** Factors influencing helminth infections in Virunga Massif (dataset 2: nest samples from Volcanos and Virunga National Parks from wet and dry season).

Effect	*d.f.*	Strongylids	*P value*	Tapeworms	*P value*
		*Chi_square*		*Chi_square*	
Area	3	376.3	< 0.001	8.6	0.03
Age/sex	3	41.6	< 0.001	21.6	< 0.001
Group size	1	6.1	0.01	15.2	< 0.001
Season	1	0.04	0.85	0.07	0.79
Area: age/sex	9	15.0	0.09	15.2	0.09
Area: group size	3	20.0	< 0.001	14.0	0.003

**Figure 5:**
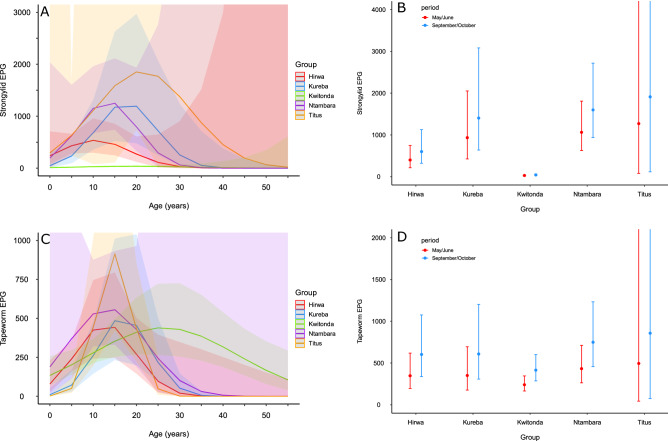
Models’ predicted mean values with 95% confidence intervals for the factors affecting helminth infections in individually sampled mountain gorillas in Virunga Massif (dataset 3). (**A**) strongylid eggs per gram (EPG)—group identity and age effects, (**B**) strongylid EPG—sampling period (season) and group identity effects, (**C**) tapeworm EPG—group identity and age effects, (**D**) tapeworm EPG—sampling period (season) and group identity effects.

In dataset 3, EPG values differed among groups (Wald’s Chi-square = 323.9, df = 4, p < 0.001; Table 4) with Kwitonda group (from S_M sector) showing lower EPG values compared to any other group (Wald’s Chi-square test, all p < 0.001), while the other groups had similar values (Fig. 5A, Table S1). There was a quadratic age effect (Wald’s Chi-square = 6.8, df = 2, p = 0.03) on strongylid EPG values with a peak occurring between 10 and 30 years (Fig. 5A). Gorillas showed higher EPG values in September/October (wet season) (estimated marginal mean ± SE = 645 ± 197 EPG) compared to May/June (boundary wet/dry season) (429 ± 130; Wald’s Chi-square = 41.3, df = 1, p < 0.001; Fig. 5B).

**Table 4:** Factors influencing helminth infections in the individual samples from five selected groups from Volcanos National Park collected in wet and dry season.

Effect	*d.f.*	Strongylids	*P value*	Tapeworms	*P value*
		*Chi_square*		*Chi_square*	
Age^2^	2	6.8	0.03	9.2	0.01
Group ID	4	323.9	< 0.001	16.8	0.002
Season	1	41.3	< 0.001	29.6	< 0.001
Sex	1	1.8	0.18	0.8	0.38
Age^2^: group ID	8	11.7	0.17	40.9	< 0.001
Age^2^: sex	2	2.4	0.31	3.1	0.21
Group ID: sex	4	0.4	0.98	7.5	0.11

### Tapeworm infections

In dataset 1, there was interactive effect of area with both group size (Wald’s Chi-square = 10.8, df = 4, p = 0.03) and age/sex class (Wald’s Chi-square = 32.1, df = 12, p = 0.001) on tapeworm EPG values (Table 2, Fig. 3B,C). Specifically, the EPG values decreased with increasing group size in K_V (estimate =  − 0.11 ± 0.03; z =  − 4.3, p < 0.001) and M area (estimate =  − 0.08 ± 0.03; z =  − 2.3, p = 0.02; Fig. 3B) but no relationship was found in other areas. Differences in EPG values between areas were detected only between K_V and BINP (Wald’s Chi-square = 10.0, df = 1, p = 0.002), with higher values in BINP. There was a significant interaction of area and age/sex on EPG values, where K_V area showed a non-significant tendency to have lower EPG values in infants than in other age/sex classes while other areas had rather similar EPG values across age/sex classes (Fig. 3C).

In dataset 2, we found an interactive effect of area and group size (Wald’s Chi-square = 14.0, df = 3, p = 0.003; Table 3, Fig. 4C). The EPG values decreased with increasing group size in the K_V area (estimate =  − 0.12 ± 0.02; z =  − 5.2, p < 0.001) but no changes in EPG values with group size were observed in other areas (Fig. 4C). EPG values in K_V area differed significantly from S_M (Wald’s Chi-square = 5.1, df = 1, p = 0.02) and V_S areas (Wald’s Chi-square = 6.5, df = 1, p = 0.01; Fig. 4C, Table S1). Similar to strongylid EPG values, infants had lower EPG values for tapeworms compared to any other age/sex classes (Wald’s Chi-square test, all p < 0.01), while the egg counts between other classes were similar (Fig. 4D, Table S1).

In dataset 3, there was an interactive effect of quadratic age and group identity on EPG values (Wald’s Chi-square = 40.9, df = 8, p < 0.001; Table 4). In particular, EPG values peaked between the age of 10 and 20 years in groups Titus, Hirwa and Kureba with the highest peak found in Titus group (Fig. 5C, Tables 4 and S1). Kwitonda group had the lowest maximum EPG value with the latest peak (age = 26 years). Individuals had higher EPG values in September/October (wet season) (estimated marginal mean ± SE = 625 ± 97 EPG) compared to May/June (boundary wet/dry season) (361 ± 97 EPG; Wald’s Chi-square = 37.6, df = 1, p < 0.001; Fig. 5D).

## Discussion

The majority of mountain gorilla fecal samples examined were positive for both strongylid nematodes and anoplocephalid tapeworms, which is in accordance with previous studies of mountain gorilla gastrointestinal (GIT) parasites^38,52,53,57^. The overall highest strongylid egg counts were recorded in the gorilla groups in the BINP, where individual cases of weight loss and poor body condition have been documented, followed by the groups from the Visoke–Sabyinyo area (V_S) of the VM. Gorilla groups ranging in the Karisimbi–Visoke area (K_V), where the highest number of severe gastritis in the VM have been documented, had notably high strongylid EPG values, especially when compared with the groups ranging in the Mikeno (M in in DRC) and the Sabyinyo–Muhavura (S_M) area of the VM, which exhibited substantially lower egg counts and no fatal GIT diseases in recent years (MGVP, unpublished data). Strongylid egg counts increased with decreasing group size in the S_M area (without GIT problems) and we did not observe higher helminth infections in silverbacks compared to other age/sex classes, which does not support our predictions. On the other hand, according to our prediction tapeworm infection intensities increased with decreasing group size in an area with high occurrence of GIT problems (K_V) but also in an area with no GIT problem (M). The observed differences in helminth infection rates among sampling areas thus only partially reflect occurrence of detected GIT problems in mountain gorillas.

Mutual effects of different population growth of VM mountain gorilla subpopulations and the BINP population, differences in the social structure particularly in VM areas, as well as differences in habitat characteristics (vegetation types interlinked with altitude) across the distribution range of mountain gorillas may explain observed differences in strongylid infections among areas. Unfortunately, information about current gorilla densities across the sampled areas are lacking, but different population dynamics have been observed in the VM areas with low strongylid egg counts (see M and S_M in Fig. 4A) in comparison to the other VM areas. For example, the VM population decline between 1960 and 1973 was driven by a severe decrease in the M (Mikeno) subpopulation in DRC (67%), accounting for 85% of the total VM decline, while in the other VM areas the population decline was much lower^1^. Since the 1980s the number of gorillas in M either declined again^7^ or did not significantly increase^4,6^. Considerably less gorillas have been found historically in S_M than in other areas of the VM and the size of this subpopulation has been stable for years^1,3,4,6,7^. In contrast, in the central areas of VM where strongylid egg counts were high (e.g. in K_V and V_S), the increase in gorilla numbers and groups since the 1980s has been the most significant^1,3,4,7,11^.

Our results thus indirectly suggest that high growth rates of gorilla subpopulations in specific VM areas in the last 40 years can be linked to high strongylid infection intensities found in these areas today. Existing data from BINP does not allow comparisons of population changes between VM and BINP, but although differences in strongylid egg counts among groups in BINP were observed (see Supplementary Table S1), we did not detect any groups with apparent low EPG, as was observed in some areas of VM (M and S_M areas). However, groups from the northern BINP area, which is characterized by lower gorilla and group densities as well as medium-altitude moist evergreen forest^9^, were not included in this study and thus should be investigated in future sampling efforts. Comparisons of our results with previous studies conducted in BINP are difficult due to significant differences in employed quantification methods. However, egg counts reported in previous studies were also high, exceeding 1000 and reaching values up to 11,000 EPG even in formalin-fixed fecal samples, where lower egg counts are always observed due to fixation^38,53,54^. Comparison of strongylid egg counts between both mountain gorilla populations and conclusions on observed high strongylid egg counts in BINP should be done with caution, as BINP is characterized by different vegetation linked to different environmental factors, presence of different sympatric non-human primates^9^ and thus the structure of the strongylid communities could differ between both gorilla populations. The very high egg counts in BINP groups and observed clinical cases of chronic weight loss and declining body condition, which may be attributable at least in part to helminth infection intensities warrant further and more detailed research of helminth infections across BINP including precise strongylid identification. In general, further studies with detailed information about current gorilla and group densities across the whole VM and BINP are urgently needed to fully understand the ability of helminths, in particular strongylids, to regulate the growth of mountain gorilla populations.

Observed relatively high strongylid EPG values and the increase of tapeworm EPG values with decreasing group size in K_V may be associated with the disintegration of a few larger multi-male groups after 2006, which led to high group densities, a higher proportion of one‐male groups and groups below average size in this area^12,13^. One-male groups, which are often smaller in size, are more vulnerable to attacks from external males, which can cause lethal wounds in adult males and infants^16,17^. Therefore, higher home range overlaps, the higher rates of interaction between groups in K_V, which can be extremely stressful events^18^, may have compromised the immune system of the gorillas, in particular the small and one-male groups, leading to an increased disease susceptibility, probable higher parasite infection intensities, and subsequently to impairing health^58^. Gorilla social structure has not been studied in detail in other areas, however census results suggest a gradual increase in the number of gorilla groups in V_S (with the highest strongylid egg counts) area since 2000^3,4,6,7^, which may have resulted in similar conditions as documented in K_V^13^ leading to high strongylid EPG values.

Marked differences in the vegetation cover (changing with elevation) that dominate gorilla habitat in our studied VM areas may also in part explain observed strongylid EPG differences among these areas. The gorilla groups with lower strongylid EPG values in the M and the S_M area predominantly utilize lower altitude forest (mixed forest) habitat, while the groups with higher strongylid EPG values in K_V and V_M exclusively range in vegetation zones at higher altitudes, including the bamboo zones, *Hagenia – Hypericum*, and subalpine^5,59^. Moreover, the recent increase in group densities in K_V^12^ also caused more groups in this area to routinely range at extremely high elevations above 3300 m (Dian Fossey Gorilla Fund—DFGF, unpublished data). In addition, the strongylid infections could be affected by gorilla diet, particularly by the consumption of medicinal plants^60^, which may vary in their availability across the VM vegetation zones. Very little is known about self-medication or even on diet composition in less studied VM areas (M, S_M). Comprehensive studies on the survival of strongylid larvae in different vegetation/elevation zones in VM are needed to better understand the impact of factors interlinked to vegetation cover on infection intensities. We cannot rule out that VM gorillas living in the lower altitude mixed forest with higher tree densities and more food in trees^11^ are more arboreal than those ranging in the other higher vegetation zones with lower tree densities, which may reduce their contact with infective larvae and result in lower strongylid intensities.

In our study, the strongylid infection intensities were lower only in infants compared to other broad age/sex classes^3^. Silverbacks, which have been diagnosed more frequently with fatal gastritis, did not exhibit the highest strongylid infections as predicted. The dataset composed of samples from individually-identified gorillas revealed no differences in helminth infections between males and females and that helminth infections in VM gorillas exhibited a convex (Type III) age-parasite intensity profile^21^, with the peak of infections around the age of 10–20 years for tapeworms and 10–30 years for strongylids. The youngest and the oldest animals had the lowest egg counts. There are several mechanisms that might account for convex age-intensity curves, including parasite-induced host mortality, acquired immunity, age-related changes in susceptibility to infection and age-dependent changes in exposure to parasites^39^. If acquired immunity influences helminth infections, than a negative correlation between infection level peak of infection and the age at which the peak occurs is expected—a phenomenon known as the ‘peak shift’ (see^23,61^). However, we observed different trends in the relationship of age and helminth egg counts and we suggest that other factors may be the drivers of the age-related helminth infection dynamics in VM gorillas. Namely, parasite-induced host mortality may be responsible for the convex (Type III) age-helminth intensity profile in mountain gorillas, which should be a focus for further study. Studies have shown that host-acquired immunity seems to be less effective at controlling strongylid infections^62,63^, while in equids age-related exposure to tapeworm infection may be an important determinant of acquired immunity and up-to-date results do not preclude the development of acquired immunity^64^.

Seasonal effects on both helminth infections were observed only when comparing helminth egg counts from May/June to those from September/October in individually sampled gorillas. We found that both strongylid and tapeworm EPG values were higher in September/October (wet season), which is in accordance with many studies in non-human primates as strongylid larvae have better conditions for survival and development during the wet season (e.g.^35,38^). In anaplocephalid tapeworms, epidemiological studies demonstrated significant seasonality with high infections linked to months with high humidity and moderate temperatures^65^.

Extreme conservation efforts have led to the recovery of the mountain gorilla, but our study points to new challenges emerging as possible “side effects” of this remarkable conservation success. Unraveling the patterns of parasite infections in a population, evaluating host exposure to infective parasite stages and susceptibility to infection and its consequences on host health is important for the conservation and survival of endangered animal species^21,66^. Small, isolated populations with low genetic diversity such as the mountain gorillas^67^ are particularly sensitive to pathogens as heterozygosity levels are linked directly to reduced population fitness via inbreeding depression^68^. The growing gorilla population has limited spatial expansion opportunities and those areas will be reaching their carrying capacity. Our study highlights that any results originating from the groups in K_V, which have been intensively studied since 1967, and from which most of our existing knowledge of the Virunga gorilla population stems, may not be applicable to the whole VM population and thus research efforts should be further expanded to groups across the VM including those ranging in the Democratic Republic of the Congo (M area). It is important to mention that strongylid nematodes in large terrestrial herbivores, such as great apes, occur in complex communities and identification of species within those communities using traditional coproscopic or even conventional molecular methods is not possible. Therefore, a strain-level identification of strongylids infecting gorillas using a metabarcoding approach is urgently warranted^69^, as the gorillas from the areas with increased incidence of GIT disease may host different communities of strongylids in comparison to other areas.

## Material and methods

### Study sites

The study was carried out across the entire range of the mountain gorilla including: (1) the Virunga Massif (VM, 451 km^2^), which spans three national parks—Volcanoes National Park (VoNP) in northwestern Rwanda, the Mikeno sector of the Virunga National Park (ViNP) in the North Kivu Province, eastern DRC and Mgahinga Gorilla National Park (MGNP) in southwestern Uganda; and (2) Bwindi Impenetrable National Park (BINP, 331 km^2^) located in southwestern Uganda. Sarambwe Reserve, a small protected area in DRC that is contiguous with BINP, was excluded for logistical reasons (Fig. 1). There are two rainy seasons (March–May and September–November) and two dry seasons (December–February and June–August) in the study region. VM is characterized by an altitude ranging from 2000 to 4500 m^70^, with different vegetation types mainly determined by elevation, including the alpine zone (above 3600 m), sub-alpine zone (3200–3600 m), *Hagenia*–*Hypericum* zone (2800–3200 m), lower altitude forest (mixed forest) (1600–2500 m), bamboo zone (2500–2800 m), disturbed woodland (2300–2800 m), open grassland and swamp (occurring in various altitudes)^55^ (Fig. 1). The most abundant vegetation type in ViNP is the mixed forest, while in VoNP it is bamboo, followed by *Hagenia—Hypericum* woodland^55^. Historically, most of the mixed forest in VoNP was destroyed by human activities, especially in the 1950s and 1960s, which pushed the gorilla population on the Rwandan side to higher elevations, where temperatures can drop to 0 °C. A single small section of lower altitude forest remains between Mt. Gahinga and Sabyinyo (the S_M). MGNP is dominated by mixed forest and bamboo^55^ (Fig. 1). The vegetation in BINP is classified as medium‐altitude moist evergreen forest and high‐altitude submontane forest. The altitude in BINP ranges from 1160 to 2607 m^56^.

### Sample collection

In 2018 fresh fecal samples were collected from habituated mountain gorillas across the VM and BINP (without northern part). Study groups in Bwindi predominantly ranged in the high‐altitude submontane forest. In the morning, feces were collected from gorilla nests of the previous night, put into plastic bags, labeled with age/sex class (silverback > 12 years, adult > 8 years, subadult/juvenile > 3.5–8 years, infant 0–3.5 years) based on the estimation of the size of fecal lobes^3,71^. Groups from ViNP and VoNP (VM) were sampled twice, once in the dry season (January/February) and once in the wet season (September/October), to assess the impact of season (sampling period) on helminth infection intensities. To investigate the impact of age and sex as host factors more accurately than estimating age/sex classes from nest samples, individual samples were also collected during focal follows in five habituated gorilla groups in VoNP in VM in May/June and in September/October (Table 1).

The groups were assigned to particular areas in VM (see above for division) based on 2018 GPS points recorded daily by staff of MGVP, Rwanda Development Board (RDB) and DFGF in Rwanda and by ViNP and MGVP in DRC (Table 1; Table S1; Fig. 5). Data on group size (number of individuals), exact age, and sex of individually sampled gorillas were obtained from DFGF, MGVP, VoNP/RDB, ViNP, MGNP, BINP and Uganda Wildlife Authority databases. Fecal samples were transported to the MGVP laboratories in Musanze (Rwanda), Kisoro (Uganda) and Goma (DRC), stored in a refrigerator, and examined within a maximum of 48 h from estimated defecation.

### Parasite quantification

The number of parasite eggs shed in feces (egg counts or eggs per gram; EPG) can be used as a proxy for nematode infection intensities in both domestic and wild animals, including non-human primates (e.g.^72,73^). Although the numbers may be influenced by individual and temporal changes in egg shedding^74,75^, the relationship between egg counts and the number of adult worms in the host has been repeatedly confirmed^25,26^. In anoplolocephalid tapeworms this relationship can be more complicated; terminated gravid proglottids detach from an adult and can either disintegrate along their route leading to the release of eggs in feces, or proglottids are shed intact. However, based on studies in horses^76,77^, we also implemented egg counts as a proxy for the quantification of tapeworm infections in gorillas.

From the inside of each fecal bolus we subsampled 3 g of feces, homogenized it in water, strained the suspended feces through a sieve into a 50 ml tube and centrifuged the mixture at 2500 rpm for 5 min. After decanting the supernatant, we quantified the strongylid and tapeworm eggs in the remaining sediment using the Mini-FLOTAC device^78^. Specifically, we resuspended the sediment in a saturated salt solution (NaCl, specific gravity = 1.2) to a total volume of 45 ml and filled both chambers of the Mini-FLOTAC using disposable Pasteur pipettes. After 10 min, the Mini-FLOTAC was read under a light microscope using 10× magnification. We counted all tapeworm and strongylid eggs and calculated eggs per gram of feces (EPG) using the following formula: EPG = total egg count in two chambers × 7.5.

### Statistical analyses

We investigated factors influencing helminth infection intensities using univariate generalized linear mixed models (GLMMs) with strongylid and tapeworm fecal EPGs as response variables. With respect to research aims, we analyzed the EPGs in three different datasets: (1) dataset 1 (n = 392); (2) dataset 2 (n = 491); and (3) dataset 3 (n = 450) (Table S1).

In datasets 1 and 2, sampling area (categorical), group size (continuous; number of individuals) and age/sex class (categorical; "infants", "juvenile/subadults", "adults", "silverbacks") were considered as explanatory variables. In dataset 2, period (binary; season: "January/February" and "September/October") was included as an additional explanatory variable. We also included the interaction of sampling area with age/sex class or group size, respectively in both dataset 1 and 2. We could not rule out repeated sampling of an individual when using nest samples because a gorilla may build and use more than one nest per night^79^. Therefore, we included age/sex class nested within group identity as random effect in all models using datasets 1 and 2 to minimize the risk of Type I error. Therefore, the probability of considering the same sample as independent was lower^80^. In dataset 3, period (season), group identity (categorical), sex (binary) and age (continuous; in years) were considered as explanatory variables. To include possible quadratic effects of age on helminth egg counts, its second-order polynomial effect was included. We further included interactions of group identity with polynomial age or sex, and the interaction of polynomial age with sex. GLMMs applied on the dataset 3 included individual identity as a random factor to account for the non-independence of repeated samples collected from the same individual.

We applied a negative binomial distribution to all models to account for the aggregated distribution of parasites within-host populations. Models were fitted with the function glmer.nb of the package “lme4” (version 1.1-23^81^) in R 3.5.0^82^. We validated each model fit using the variance inflation factor to assess multicollinearity, visual inspection of the model residuals, homoscedasticity of each variable residuals, random effect quantiles Q–Q plots and found them satisfactory. We presented and interpreted results from the full model including all the variables and their interactions or polynomials for which we made predictions in the introduction. This model approach allowed us to test our hypotheses and reduce Type I error and problems of biased effect sizes^83^. However, main effects are generally not biologically interpretable if involved in interactions or polynomial terms^84^. Thus, to enable interpretation of main effects in the presence of interactions or polynomials, we refitted each model with manually re-coded categorical variables (i.e. constructing dummy variables) and then centered (subtracted the sample mean from all the variable values) all the independent variables entering the full model (e.g.^85,86^). Significance tests for fixed effects were performed using Type II Wald's Chi-square tests under which we compared a focal dummy variable against remaining dummy variables. This was accomplished by setting the focal dummy variables as the reference (e.g. “infant” and “M” dummy variables were assigned with value 0) in the model structure. To compare/test all the relevant combinations, we re-fitted the model with a slightly modified model structure where we only changed the reference dummy variables. Albeit the full models allowed us to address predictions, we also wanted to identify biologically most relevant factors by finding model best fitting the data^87^. Thus, we further performed a complementary analysis to the best models using model selection procedure according to the Akaike information criterion corrected for small sample sizes (AIC_c_)^88^ with R package MuMIn^89^. Best models and their outputs were presented in the Supplementary material.

### Ethics approval

This research involved only non-invasive work with wild non-human primates. All work was done in accordance with guidelines of the national authorities.

## Supplementary Information

Supplementary Information 1.

## Data Availability

Data are available in the Figshare repository https://figshare.com/s/61461cfcb8d9657c3bcb.
