# Supplementary material for: Heterogeneity in patterns of helminth infections across populations of mountain gorillas (Gorilla beringei beringei)
Source: Sci Rep. 2021 May 25;11:10869. doi: 10.1038/s41598-021-89283-4 (PMC8149636; doi:10.1038/s41598-021-89283-4)
Supplement: Supplementary file 1 — Supplementary Information 1. [file 41598_2021_89283_MOESM1_ESM.docx]

**Heterogeneity in patterns of helminth infections across populations of mountain gorillas (*Gorilla beringei beringei)***

Authors

Klara J. Petrželková*, Carine Uwamahoro, Barbora Pafčo, Barbora Červená, Peter Samaš, Antoine Mudakikwa, Richard Muvunyi, Prosper Uwingeli, Kirsten Gilardi, Julius Nziza, Jean Bosco Noheri, Winnie Eckardt, Felix Ndagijimana, Benard Ssebide, Ricky Okwirokello, Fred Nizeyimana, Eddy Kambale Syaluha, Gaspard Nzayisenga, Luis Flores Girón, Méthode Bahizi, Adrien Emile Ntwari, Jean-Paul Lukusa, Jean Claude Tumushime, Damien Mangura, Jeff Mapilanga, Arthur Kalonji, Robert Aruho, Anna Stryková, Zuzana Tehlarová, Rita Cameira, Linda Lowenstine, Jan Šlapeta, Dušan Romportl, Nicola Ferrari, Michael Cranfield and David Modrý

*corresponding author, petrzelkova@ivb.cz

**Supplementary Table 1:** Information about sampled groups and helminth infections for each dataset (A-C) in 2018. Inf – infants, Juv/Sub – juveniles/subadults, Ad – adults (blackbacks and adult females), SB **–** silverbacks; EPG - eggs per gram. Karisimbi-Visoke (K_V), Visoke-Sabyinyo (V_S), Sabyinyo-Muhavura (S_M) and Mikeno (M). Bwindi Impenetrable National Park (BINP).

A. dataset 1

| **Group Name** | **Area** | **Group size**  **(N)** | **Samples analysed**  **(N)** | **Samples by age/sex classes (N)** | | | | **Strongylid EPG** | | **Tapeworm EPG** | |
| --- | --- | --- | --- | --- | --- | --- | --- | --- | --- | --- | --- |
|  |  |  |  | Inf | Juv/Sub | Ad | SB | Median | Min /Max | Median | Min /Max |
| Bageni | M | 30 | 19 | 1 | 7 | 11 | 0 | 15 | 0-135 | 23 | 0-315 |
| Humba |  | 11 | 7 | 1 | 1 | 3 | 2 | 53 | 15-158 | 68 | 8-818 |
| Kabirizi |  | 19 | 11 | 1 | 3 | 5 | 2 | 30 | 15-128 | 38 | 0-833 |
| Lulengo |  | 11 | 8 | 2 | 0 | 4 | 2 | 16 | 0-120 | 413 | 23-5775 |
| Munyaga |  | 9 | 6 | 1 | 2 | 1 | 2 | 31 | 8-143 | 151 | 23-390 |
| Nyakamwe |  | 12 | 7 | 2 | 2 | 1 | 2 | 75 | 45-113 | 210 | 30-675 |
| Rugendo |  | 10 | 7 | 0 | 0 | 4 | 3 | 8 | 0-23 | 45 | 0-263 |
|  |  |  |  |  |  |  |  |  |  |  |  |
| Igisha | K_V | 31 | 14 | 2 | 1 | 8 | 3 | 630 | 158-2280 | 0 | 0-30 |
| Isabukuru |  | 10 | 9 | 1 | 4 | 3 | 1 | 263 | 150-998 | 158 | 0-765 |
| Isimbi |  | 20 | 8 | 0 | 0 | 7 | 1 | 638 | 105-930 | 8 | 0-968 |
| Iyambere |  | 5 | 4 | 1 | 0 | 2 | 1 | 1962 | 653-3180 | 559 | 0-2670 |
| Kubona |  | 5 | 5 | 1 | 0 | 3 | 1 | 308 | 173-810 | 45 | 0-1088 |
| Kureba |  | 8 | 7 | 2 | 0 | 3 | 2 | 1065 | 105-1995 | 270 | 0-855 |
| Mafunzo |  | 13 | 11 | 2 | 0 | 8 | 1 | 315 | 165-1238 | 150 | 0-495 |
| Musilikale |  | 20 | 12 | 3 | 1 | 5 | 3 | 556 | 23-1223 | 177 | 0-1058 |
| Pablo |  | 23 | 17 | 0 | 7 | 7 | 3 | 435 | 105-1418 | 68 | 0-488 |
| Susa |  | 18 | 16 | 3 | 1 | 8 | 4 | 758 | 383-1245 | 87 | 0-300 |
| Titus |  | 6 | 5 | 1 | 1 | 1 | 2 | 480 | 180-698 | 128 | 8-203 |
|  |  |  |  |  |  |  |  |  |  |  |  |
| Hirwa | S_M | 16 | 17 | 4 | 7 | 4 | 2 | 120 | 15-615 | 105 | 0-930 |
| Kwitonda |  | 34 | 10 | 3 | 2 | 3 | 2 | 23 | 0-75 | 117 | 0-1238 |
| Nyakagezi |  | 9 | 8 | 1 | 1 | 4 | 2 | 19 | 0-38 | 852 | 8-2438 |
|  |  |  |  |  |  |  |  |  |  |  |  |
| Agashya | V_S | 24 | 11 | 1 | 1 | 7 | 2 | 578 | 195-3128 | 233 | 0-735 |
| Amahoro |  | 21 | 12 | 3 | 1 | 8 | 0 | 957 | 413-3870 | 274 | 0-1043 |
| Muhoza |  | 11 | 6 | 1 | 0 | 4 | 1 | 852 | 210-3053 | 282 | 188-383 |
| Ntambara |  | 12 | 9 | 0 | 2 | 6 | 1 | 638 | 173-2963 | 473 | 285-1613 |
| Sabyinyo |  | 19 | 15 | 4 | 2 | 6 | 3 | 1755 | 510-10050 | 480 | 90-4995 |
| Umubano |  | 13 | 9 | 0 | 2 | 3 | 4 | 1050 | 210-4770 | 203 | 0-593 |
|  |  |  |  |  |  |  |  |  |  |  |  |
| Bikingi | BINP | 15 | 11 | 1 | 3 | 6 | 1 | 1965 | 998-4118 | 503 | 128-1305 |
| Bitukura |  | 12 | 8 | 2 | 1 | 4 | 1 | 702 | 420-2190 | 375 | 98-878 |
| Busingye |  | 12 | 8 | 1 | 2 | 4 | 1 | 1320 | 233-3660 | 263 | 75-465 |
| Bweza |  | 11 | 8 | 1 | 0 | 4 | 3 | 811 | 165-1598 | 267 | 135-510 |
| Christmas |  | 8 | 6 | 1 | 2 | 14 | 4 | 1336 | 233-2790 | 387 | 218-2273 |
| Kahunge |  | 23 | 20 | 2 | 5 | 11 | 2 | 1366 | 0-4050 | 567 | 15-1463 |
| Kyaguliro |  | 9 | 4 | 0 | 2 | 1 | 1 | 327 | 143-458 | 233 | 143-345 |
| Mishaya |  | 10 | 7 | 1 | 1 | 4 | 1 | 803 | 38-2190 | 203 | 45-810 |
| Mucunguzi |  | 8 | 5 | 0 | 1 | 3 | 1 | 1793 | 293-3240 | 360 | 255-893 |
| Mukiza |  | 12 | 10 | 0 | 3 | 6 | 1 | 1231 | 195-2130 | 278 | 23-450 |
| Nkuringo |  | 14 | 14 | 1 | 3 | 8 | 2 | 1819 | 750-3953 | 181 | 15-968 |
| Nshongi |  | 7 | 6 | 2 | 0 | 3 | 1 | 518 | 8-1035 | 113 | 8-743 |
| Oruzogo |  | 17 | 15 | 4 | 2 | 6 | 3 | 1793 | 323-4028 | 375 | 23-968 |

B. dataset 2

| **Group Name** | **Area** | **Mean Group Size**  **(N)** | **Analysed samples**  **(N)** | **Samples by age/sex classes (N)** | | | | **1° Period**  **(January/February)** | | | | **2° Period**  **(September/October)** | | | |
| --- | --- | --- | --- | --- | --- | --- | --- | --- | --- | --- | --- | --- | --- | --- | --- |
|  |  |  |  |  |  |  |  | **Strongylid EPG** | | **Tapeworm EPG** | | **Strongylid EPG** | | **Tapeworm EPG** | |
|  |  |  |  | Inf | Juv/Sub | Ad | SB | Median | Min / Max | Median | Min /Max | Median | Min / Max | Median | Min / Max |
| Bageni | Mikeno | 31 | 42 | 3 | 10 | 26 | 3 | 27 | 0-240 | 196 | 0-510 | 15 | 0-135 | 19 | 0-173 |
| Humba |  | 11 | 10 | 1 | 2 | 4 | 3 | 49 | 30-68 | 45 | 8-83 | 61 | 15-158 | 72 | 23-818 |
| Kabirizi |  | 19 | 27 | 2 | 4 | 15 | 6 | 57 | 0-173 | 139 | 0-900 | 30 | 15-128 | 38 | 0-833 |
| Lulengo |  | 11 | 17 | 5 | 2 | 7 | 3 | 4 | 0-30 | 199 | 8-1328 | 8 | 0-120 | 420 | 23-5775 |
| Munyaga |  | 9 | 12 | 1 | 4 | 3 | 4 | 68 | 8-285 | 154 | 30-248 | 31 | 8-143 | 151 | 23-390 |
| Nyakamwe |  | 12 | 18 | 3 | 3 | 6 | 6 | 23 | 0-435 | 135 | 0-435 | 75 | 45-113 | 210 | 30-675 |
| Rugendo |  | 10 | 16 | 1 | 1 | 7 | 7 | 31 | 0-83 | 267 | 0-735 | 8 | 0-23 | 57 | 0-263 |
|  |  |  |  |  |  |  |  |  |  |  |  |  |  |  |  |
| Igisha | K_V | 29 | 30 | 3 | 3 | 15 | 9 | 582 | 143-1215 | 15 | 0-113 | 631 | 158-2280 | 0 | 0-30 |
| Isabukuru |  | 12 | 18 | 2 | 7 | 8 | 1 | 1253 | 570-1980 | 285 | 83-780 | 263 | 150-998 | 158 | 0-765 |
| Isimbi |  | 19 | 17 | 2 | 0 | 13 | 2 | 375 | 68-1680 | 15 | 0-173 | 638 | 105-930 | 8 | 0-968 |
| Iyambere |  | 6 | 9 | 2 | 0 | 5 | 2 | 1325 | 533-2078 | 311 | 0-2048 | 1020 | 150-3660 | 345 | 0-2670 |
| Kubona |  | 4 | 8 | 1 | 0 | 5 | 2 | 893 | 825-998 | 315 | 23-1485 | 308 | 173-810 | 45 | 0-1088 |
| Kureba |  | 8 | 14 | 3 | 1 | 7 | 3 | 660 | 195-3750 | 270 | 60-1260 | 1065 | 105-1995 | 270 | 0-855 |
| Musilikale |  | 19 | 23 | 5 | 6 | 6 | 6 | 480 | 75-908 | 98 | 15-795 | 556 | 23-1223 | 177 | 0-1058 |
| Pablo |  | 23 | 33 | 3 | 8 | 18 | 4 | 795 | 323-1515 | 113 | 0-1538 | 495 | 105-2528 | 79 | 0-488 |
| Susa |  | 18 | 28 | 5 | 1 | 15 | 7 | 548 | 323-1215 | 8 | 0-443 | 709 | 210-1230 | 87 | 0-300 |
| Titus |  | 7 | 15 | 3 | 2 | 5 | 5 | 1275 | 300-2933 | 199 | 0-1193 | 480 | 180-698 | 128 | 8-203 |
|  |  |  |  |  |  |  |  |  |  |  |  |  |  |  |  |
| Hirwa | S_M | 17 | 31 | 5 | 11 | 11 | 4 | 366 | 53-1725 | 150 | 0-750 | 120 | 15-615 | 128 | 15-615 |
| Kwitonda |  | 32 | 23 | 6 | 2 | 13 | 2 | 23 | 0-210 | 150 | 0-1103 | 23 | 0-75 | 19 | 0-75 |
|  |  |  |  |  |  |  |  |  |  |  |  |  |  |  |  |
| Agashya | V_S | 23 | 26 | 1 | 4 | 18 | 3 | 297 | 150-2115 | 390 | 15-653 | 586 | 195-6308 | 244 | 0-735 |
| Muhoza |  | 10 | 14 | 1 | 1 | 10 | 2 | 510 | 195-2445 | 334 | 75-510 | 852 | 210-3053 | 281 | 188-383 |
| Ntambara |  | 12 | 19 | 1 | 6 | 9 | 3 | 930 | 150-1590 | 387 | 203-615 | 593 | 150-2963 | 454 | 285-1613 |
| Sabyinyo |  | 18 | 24 | 3 | 5 | 13 | 3 | 240 | 150-1245 | 188 | 0-398 | 1440 | 150-6405 | 480 | 90-4995 |
| Umubano |  | 13 | 17 | 0 | 3 | 8 | 6 | 488 | 150-2415 | 173 | 30-945 | 14440 | 210-4770 | 192 | 0-593 |
|  | |  |  |  |  |  |  |  |  |  |  |  |  |  |  |

C. dataset 3

| **Group Name** | **Area** | **Mean Group Size**  (N) | **Samples Analysed**  (N) | **Mean Age**  (min-max) | **Sex**  (M/F) | **Strongylid EPG** | | **Tapeworm EPG** | |
| --- | --- | --- | --- | --- | --- | --- | --- | --- | --- |
|  |  |  |  |  |  | Median | Min /Max | Median | Min /Max |
| Hirwa | S_M | 16 | 99 | 10.4 (0.4-22.2) | 34/65 | 368 | 8-1275 | 248 | 0-1590 |
| Kureba | K_V | 8 | 52 | 15.1 (3.7-32.2) | 26/26 | 436 | 113-2175 | 192 | 0-1658 |
| Kwitonda | S_M | 31 | 171 | 16.5 (2.6-52.0) | 81/90 | 23 | 0-818 | 203 | 0-8190 |
| Ntambara | V_S | 12 | 76 | 13.7 (1.8-20.6) | 41/35 | 919 | 105-4673 | 402 | 0-2490 |
| Titus | K_V | 7 | 52 | 14.4 (2.4-22.7) | 35/17 | 1365 | 210-3263 | 308 | 0-1493 |

**Supplementary Table 2:** Outputs of (A) model selection and (B) best model testing effects on helminth infections in the dataset 1 (nest samples collected in a single period/season across the whole Virunga Massif and Bwindi Impenetrable National Park). Model selection was based on Akaike information criterion corrected for small sample sizes (AIC_c_) and shown are the best five models together with the full model at the bottom for comparison. Number of parameters (df), model log-likelihood (LL), corrected AIC criterion (AIC_c_), the difference between AIC_c_ of the focal model and the best model (ΔAIC_c_) and Akaike weight (weight; the relative likelihood of a model) for each of the five models are shown. Full versus best model comparison was marginally non-significant for strongylids (Wald’s Chi-square = 26.7, df = 17, p = 0.061) and significant for tapeworms (Wald’s Chi-square = 25.8, df = 15, p = 0.041).

(A)

| **Variables** | **d.f.** | **LL** | **AIC_c_** | **ΔAIC_c_** | **weight** |
| --- | --- | --- | --- | --- | --- |
| ***Strongylids*** |  |  |  |  |  |
| Age/sex + Area | 11 | −2797.7 | 5618.2 | 0.00 | 0.503 |
| Age/sex + Area + Age/sex:Area | 23 | −2785.3 | 5619.7 | 1.52 | 0.236 |
| Age/sex + Area + Group size | 12 | −2797.7 | 5620.3 | 2.11 | 0.176 |
| Age/sex + Area + Group size + Age/sex:Area | 24 | −2785.3 | 5621.9 | 3.77 | 0.076 |
| Age/sex + Area + Group size + Area:Group size | 16 | −2796.8 | 5627.0 | 8.81 | 0.006 |
| Full model | 28 | −2784.0 | 5628.5 | 10.40 | 0.003 |
| ***Tapeworms*** |  |  |  |  |  |
| Area + Group size + Area:Group size | 13 | −2532.6 | 5092.1 | 0.00 | 0.694 |
| Area + Group size | 9 | −2538.5 | 5095.4 | 3.30 | 0.134 |
| Age/sex + Area + Group size + Area:Group size | 16 | −2531.9 | 5097.2 | 5.03 | 0.056 |
| Group size | 5 | −2543.7 | 5097.6 | 5.46 | 0.045 |
| Age/sex + Area + Group size + Age/sex:Area | 24 | −2523.7 | 5098.6 | 6.47 | 0.027 |
| Full model | 28 | −2519.2 | 5098.9 | 6.82 | 0.023 |

(B)

| Effect |  | Strongylids |  |  | Tapeworms |  |
| --- | --- | --- | --- | --- | --- | --- |
|  | *d.f.* | *Chi_square* | *P value* |  | *Chi_square* | *P value* |
| Area | 4 | 235.0 | <0.001 |  | 4 | 16.4 |
| Age/sex | 3 | 32.7 | <0.001 |  | – | – |
| Group size | 1 | – | – |  | 1 | 20.9 |
| Area : Age/sex | 12 | – | – |  | – | – |
| Area : Group size | 4 | – | – |  | 4 | 14.8 |

**Supplementary Table 3:** Outputs of (A) model selection and (B) best model testing effects on helminth infections in the dataset 2 (nest samples from Volcanoes National Park and Virunga National Park collected in two periods/seasons). Model selection was based on Akaike information criterion corrected for small sample sizes (AIC_c_) and shown are the best five models together with the full model at the bottom for comparison. Number of parameters (df), model log-likelihood (LL), corrected AIC criterion (AIC_c_), the difference between AIC_c_ of the focal model and the best model (ΔAIC_c_) and Akaike weight (weight; the relative likelihood of a model) for each of the five models are shown. * The model is identical to the full model. Full versus best model comparison was non-significant for strongylids (Wald’s Chi-square = 14.9, df = 10, p = 0.14) and marginally non-significant for tapeworms (Wald’s Chi-square = 18.0, df = 10, p = 0.054).

(A)

| **Variables** | **d.f.** | **LL** | **AIC_c_** | **ΔAIC_c_** | **weight** |
| --- | --- | --- | --- | --- | --- |
| ***Strongylids*** |  |  |  |  |  |
| Age/sex + Area + Group size + Area:Group size | 14 | −3278.3 | 6585.4 | 0.00 | 0.598 |
| Age/sex + Area + Group size + Period + Area:Group size | 15 | −3278.3 | 6587.5 | 2.13 | 0.206 |
| Age/sex + Area | 10 | −3284.8 | 6590.0 | 4.59 | 0.060 |
| Age/sex + Area + Group size + Age:Area + Area:Group size | 23 | −3271.2 | 6590.8 | 5.38 | 0.041 |
| Age/sex + Area + Group size | 11 | −3284.2 | 6590.9 | 5.47 | 0.039 |
| Full model | 24 | −3271.2 | 6592.9 | 7.56 | 0.014 |
| ***Tapeworms*** |  |  |  |  |  |
| Age/sex + Area + Group size + Area:Group size | 14 | −3037.5 | 6103.9 | 0.00 | 0.388 |
| Age/sex + Area + Group size + Age:Area + Area:Group size | 23 | −3028.4 | 6105.2 | 1.30 | 0.202 |
| Age/sex + Area + Group size + Period + Area:Group size | 15 | −3037.5 | 6106.1 | 2.12 | 0.135 |
| Area + Group size + Area:Group size | 11 | −3042.4 | 6107.3 | 3.35 | 0.073 |
| *Age+Area+Group size+Period+Age:Area+Area:Group size | 24 | −3028.4 | 6107.4 | 3.46 | 0.069 |

(B)

| Effect |  | Strongylids |  |  | Tapeworms |  |
| --- | --- | --- | --- | --- | --- | --- |
|  | *d.f.* | *Chi_square* | *P value* |  | *Chi_square* | *P value* |
| Area | 3 | 386.8 | <0.001 |  | 10.3 | 0.02 |
| Age/sex | 3 | 35.5 | <0.001 |  | 12.0 | 0.007 |
| Group size | 1 | 5.3 | 0.02 |  | 15.0 | <0.001 |
| Season | 1 | – | – |  | – | – |
| Area : Age/sex | 9 | – | – |  | – | – |
| Area : Group size | 3 | 18.2 | <0.001 |  | 14.8 | 0.002 |

**Supplementary Table 4:** Outputs of (A) model selection and (B) best model testing effects on helminth infections in the dataset 3 (individual samples from five selected groups from Volcanoes National Park collected in two periods/seasons). Model selection was based on Akaike information criterion corrected for small sample sizes (AIC_c_) and shown are the best five models together with the full model in the bottom for comparison. Number of parameters (df), model log-likelihood (LL), corrected AIC criterion (AIC_c_), the difference between AIC_c_ of the focal model and the best model (ΔAIC_c_) and Akaike weight (weight; the relative likelihood of a model) for each of the five models are shown. * The model is identical to the full model. Full versus best model comparison was non-significant both for strongylids (Wald’s Chi-square = 14.3, df = 14, p = 0.43) and tapeworms (Wald’s Chi-square = 11.9, df = 7, p = 0.11).

(A)

| **Variables** | **d.f.** | **LL** | **AIC_c_** | **ΔAIC_c_** | **weight** |
| --- | --- | --- | --- | --- | --- |
| ***Strongylids*** |  |  |  |  |  |
| Age^2 + Group + Period + Sex | 11 | −2872.3 | 5767.2 | 0.00 | 0.302 |
| Age^2 + Group + Period | 10 | −2873.7 | 5767.8 | 0.62 | 0.222 |
| Group + Period | 8 | −2876.1 | 5768.5 | 1.26 | 0.161 |
| Group + Period + Sex | 9 | −2875.2 | 5768.9 | 1.64 | 0.133 |
| Age^2 + Group + Period + Sex + Age^2:Sex | 13 | −2871.1 | 5769.0 | 1.77 | 0.125 |
| Full model | 13 | −2865.1 | 5783.4 | 16.15 | 0.000 |
| ***Tapeworms*** |  |  |  |  |  |
| Age^2 + Group + Period + Age^2:Group | 18 | −3058.9 | 6155.5 | 0.00 | 0.597 |
| Age^2 + Group + Period + Sex + Age^2:Group | 19 | −3058.6 | 6157.0 | 1.56 | 0.274 |
| Age^2 + Group + Period + Sex + Age^2:Group + Age^2:Sex | 21 | −3058.2 | 6160.6 | 5.11 | 0.046 |
| Age^2 + Group + Period + Sex + Age^2:Group + Group:Sex | 23 | −3056.2 | 6161.0 | 5.51 | 0.038 |
| *Age^2 + Group + Period + Sex + Age^2:Group + Group:Sex + Age^2:Sex | 25 | −3054.7 | 6162.5 | 7.07 | 0.017 |

(B)

| Effect |  | Strongylids |  |  | Tapeworms |  |
| --- | --- | --- | --- | --- | --- | --- |
|  | *d.f.* | *Chi_square* | *P value* |  | *Chi_square* | *P value* |
| Age^2 | 2 | 2 | 5.9 |  | 2 | 10.7 |
| Group | 4 | 4 | 263.6 |  | 4 | 15.8 |
| Season | 1 | 1 | 40.0 |  | 1 | 25.4 |
| Sex | 1 | 1 | 2.8 |  | – | – |
| Age^2 : Group | 8 | – | – |  | 8 | 41.1 |
| Age^2 : Sex | 2 | – | – |  | – | – |
| Group ID : Sex | 4 | – | – |  | – | – |
